# Difficulties in the diagnosis of periapical translucencies and in the classification of cemento-osseous dysplasia

**DOI:** 10.1186/s12903-019-0843-0

**Published:** 2019-07-10

**Authors:** Andrea Brody, Attila Zalatnai, Krisztian Csomo, Andrea Belik, Csaba Dobo-Nagy

**Affiliations:** 1 Department of Oral Diagnostics, Faculty of Dentistry, Semmelweis University, Szentkirályi u. 47, Budapest, 1088 Hungary; 2 Department of Pathology and Experimental Cancer Research, Faculty of Medicine, Semmelweis University, Üllői út 26, Budapest, 1085 Hungary; 3 Department of Medical Chemistry, Molecular Biology and Pathobiochemistry, Semmelweis University, Tűzoltó u. 37-47, Budapest, 1094 Hungary

**Keywords:** Cemento-osseous dysplasia, Classification, Differential diagnosis, Familial form of COD, Florid, Misdiagnosis, Periapical translucencies

## Abstract

**Background:**

Cemento-osseous dysplasia is a benign fibro-osseous lesion of the tooth-bearing region of the jaws with a periodontal ligament origin. It appears predominantly in Black and Asian middle-aged females. Its importance is that it could mimic a periapical lesion in the early, translucent stage.

**Case presentation:**

In this report a rare case of familial cemento-osseous dysplasia is presented: a 50-years old Caucasian woman with labial paraesthesia and radiological translucency around the roots of the mandibular incisors and the first molar teeth. The lesion around the first molar was diagnosed as periapical granuloma and a root canal treatment was carried out. The diagnosis of florid cemento-osseous dysplasia and the treatment plan based on two- and three-dimensional radiographic examinations were certified histologically after surgical removal of the lesion. We screened the family members - including the patient’s mother, daughter and son - and identified a periapical version of cemento-osseous dysplasia in the daughter. Our case highlights the difficulties of differential diagnosis of cemento-osseous dysplasia and other periapical pathologies. The inconsistencies in the present classification of cemento-osseous dysplasia are also discussed with a proposal for a different classification based on new aspects that would be very helpful in setting up a correct treatment plan.

**Conclusion:**

- Differentiation of endodontic and non-endodontic origin of radiolucency and distinguishing it from anatomical landmarks by appropriate clinical evaluation and using vitality testing can give an opportunity to prevent unnecessary endodontic treatment.
- The current categories of cemento-osseous dysplasia classification do not cover the early stage of a hereditary florid form of cemento-osseous dysplasia.
- Instead of anatomical location of the lesion, clinical and genetic features may be recommended as parameters of cemento-osseous dysplasia classification.

## Background

Cemento-osseous dysplasia (COD) is an uncommon fibro-osseous lesion of the jaws with a periodontal ligament origin [1–3], which - in its early stage - can mimic a periapical lesion [4]. COD is a non-neoplastic, radiolucent and/or radiopaque, non-encapsulated, cellular-fibrotic lesion with calcified structures – such as irregular osseous trabeculae and cementoid mass - affecting the tooth-bearing area and the cancellous part of the jaws [5] associated with vital teeth usually without any clinical sign or complaint [6]. This is the reason why many cases are diagnosed accidentally during radiographic examinations which were ordered for other reasons. The disease predominantly affects middle-aged Asian and African females [7, 8], and is often only recognized in an advanced stage. It is usually not prone to growth (except some cases of the florid form) and in many cases, the lesion closes spontaneously [9]. In the last, 4th edition of the WHO Classification of Head and Neck Tumours [10], cemento-osseous dysplasia was classified in the fibro- and chondro-osseous lesions, in the group of “benign odontogenic tumours and allied lesions”. In the 2005 edition [11] it is also defined as a member of benign fibro-osseous dysplasias, but the term was changed to osseous-dysplasia without the prefix “cemento”, stemming from the consideration that the cementum and the bone tissue can only be distinguished from each other by their origin and their respective relationship to the root [4, 12]. Simply put, the mineralized concentric formation inside the lesion is referred to as cementum, while the long-shaped one as bone trabecula [3, 6]. Though as in the case of the cemento-ossifying fibroma, the 4th edition of the WHO Classification reverts to the term “cemento-osseous dysplasia” with the purpose of highlighting the odontogenic origin of COD, emanating from the undifferentiated fibroblasts of the periodontal ligaments. COD has been recently divided into three subtypes based on the anatomical location: periapical, focal and florid. The occurrence of the familial type is very rare and according to the current classification, it only presents as the florid type [2]. COD can affect only one or several quadrants, both in the mandible and the maxilla.

## Case presentation

### The first patient

**Fig. 1:**
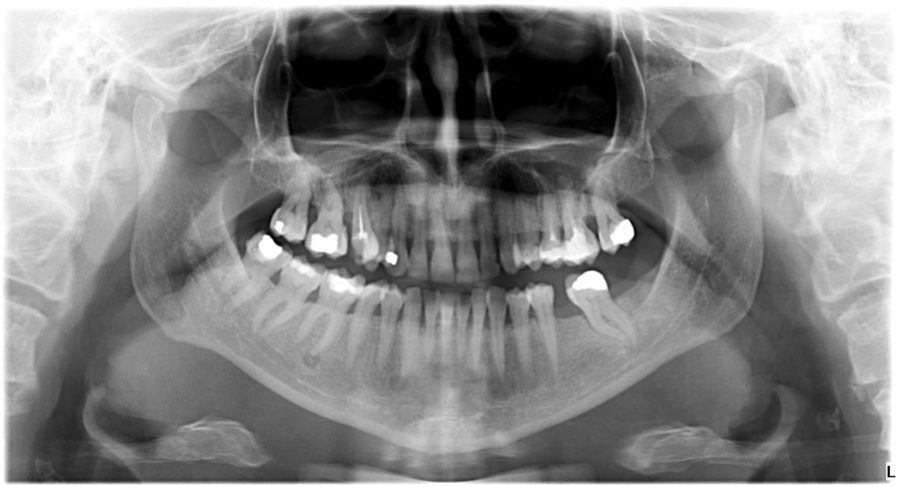
Panoramic radiograph of the first patient. Radiolucent lesions in the mandible mimicking periapical endodontic lesions around vital teeth in the canine and the right molar region

**Fig. 2:**
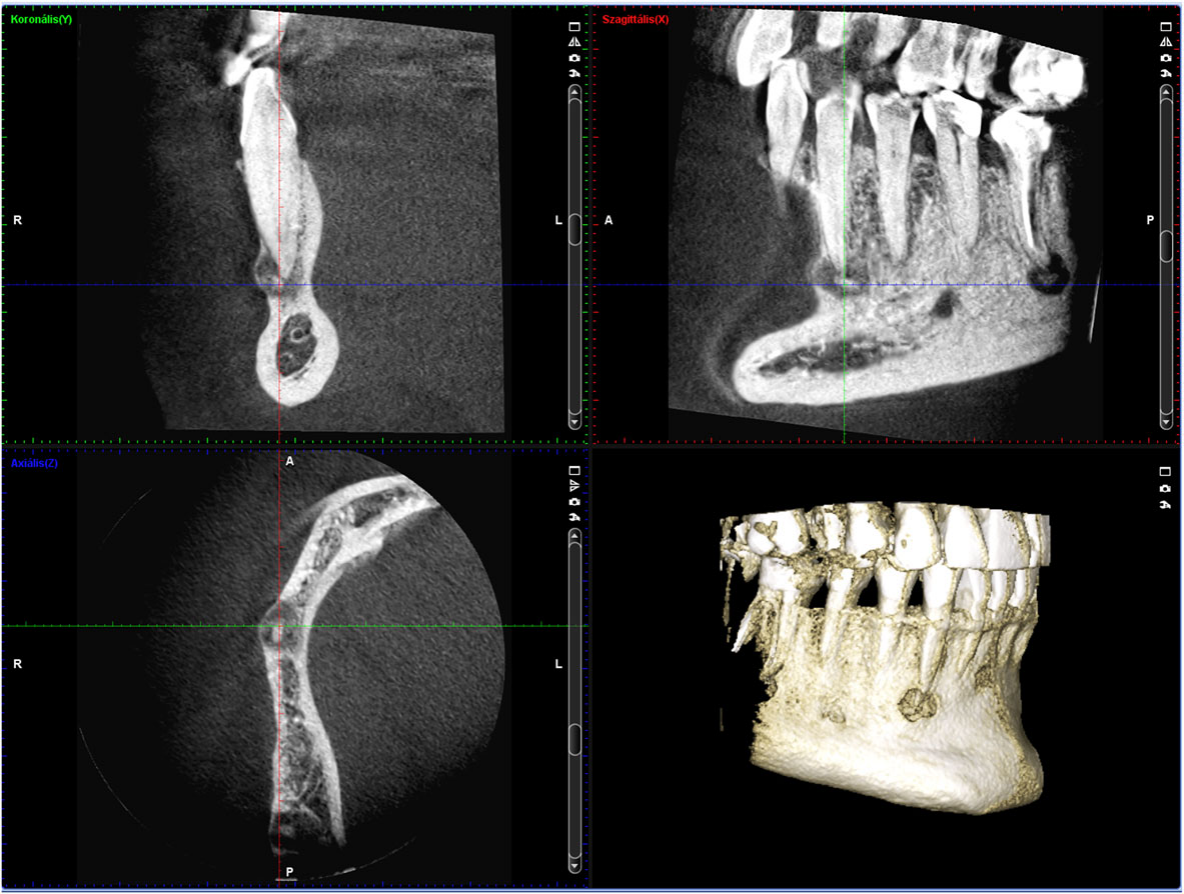
The lesion on the lower right canine extruded and extremely thinned the buccal cortical bone on CBCT sections of patient 1

A 50-year-old Caucasian female visited the regional dental office due to uncertain pain in the right mandibular region. No significant diseases were mentioned in the anamnesis. The patient was diagnosed with gingivitis, and treated with a non-steroid anti-inflammatory drug and oral rinsing with chlorhexidine. The condition of the patient did not improve significantly. When she returned a week later, she had no alleviation, but labial paresthesia on the right side of her lip. As the cause of the new symptom remained unidentified, she was referred to the oral surgery group. Initially, the paraesthesia affected only the right side of the lower lip, but by this time it had spread to the skin of the right side of her chin. The oral surgeons decided to extract the lower right wisdom tooth, but the labial paraesthesia still persisted. Since there was an extended composite restoration in the lower right first molar, and translucency around its apical region was visible, the lesion was diagnosed as periapical granuloma. The lesion on tooth 46 was not close to the mandibular canal; this can be seen on Fig. 1. Therefore, root canal treatment was carried out, despite the uncertain result of the percussion, palpation and sensibility test. The transparent lesion around the apex and the negative sensibility test lead to the decision of endodontic treatment. Diagnosis was made by a dentist who is not a staff member of our Department. The treatment revealed that the pulp was vital. Our oral diagnostic team examined the patient, and we diagnosed the case as cemento-osseous dysplasia based on the panoramic radiograph showing apical translucency lesions around the anterior teeth with vital pulp (Fig. 1). The paraesthesia has been continually present ever since. Because the lesion around the lower right canine had already involved the cortical bone by CBCT scan (Fig. 2), a root canal filling and removal of the apical lesion were performed. On the CBCT image the involvement of the entire periapical region can be seen including the lingual region and the buccal cortical (Fig. 2). It became clear during the planning of the surgery that the blood vessels and nerves of tooth 43 would be injured.

The root canal treatment was carried out on the lower right canine in a single-visit treatment using local anaesthaesia. We explored the entire root canal length using a size #15 hand instrument. The working length was determined using an apex locator, (Woodpex III, Guilin,China), then the length was also confirmed with radiographic imaging. Following the length determination, the root canal was shaped using Wave One (Densply Maillefer, York, USA) rotary instrument. The root canal was obturated using guttapercha and AH Plus sealer (Dentsply DeTrey GmbH, Konstanz, Germany) with lateral condensation technique. Glass ionomer cement (Fuji IX GP, GC Co., Tokyo, Japan) was then applied to seal off the access cavity, while the permanent restoration was done.

We prepared an intraoral mucoperiostal flap using an L-shaped incision and the surgery was carried out by using a surgical microscope (Aspheron, Schmidt and Bender Hungaria, Budapest, Hungary).

We opened up the buccal cortical bone using a surgical bur, thereafter we removed the lesion surrounding the apex of the root. The root apex was resected and a retrograde root canal filling was placed using mineral trioxid aggregate (MTA+, Cerkamed, Stalowa Wola, Poland).

The area of the lesion was augmented using gentamicin impregnated BoneAlbumin (OrthoSera Dental Zrt., Gyor, Hungary). We seeked to lower the chance of osteomyelitis occuring with the use of gentamicin. Sutures were then carefully placed to achieve tensionfree closure of the flap for optimal healing.

### The second patient

**Fig. 3:**
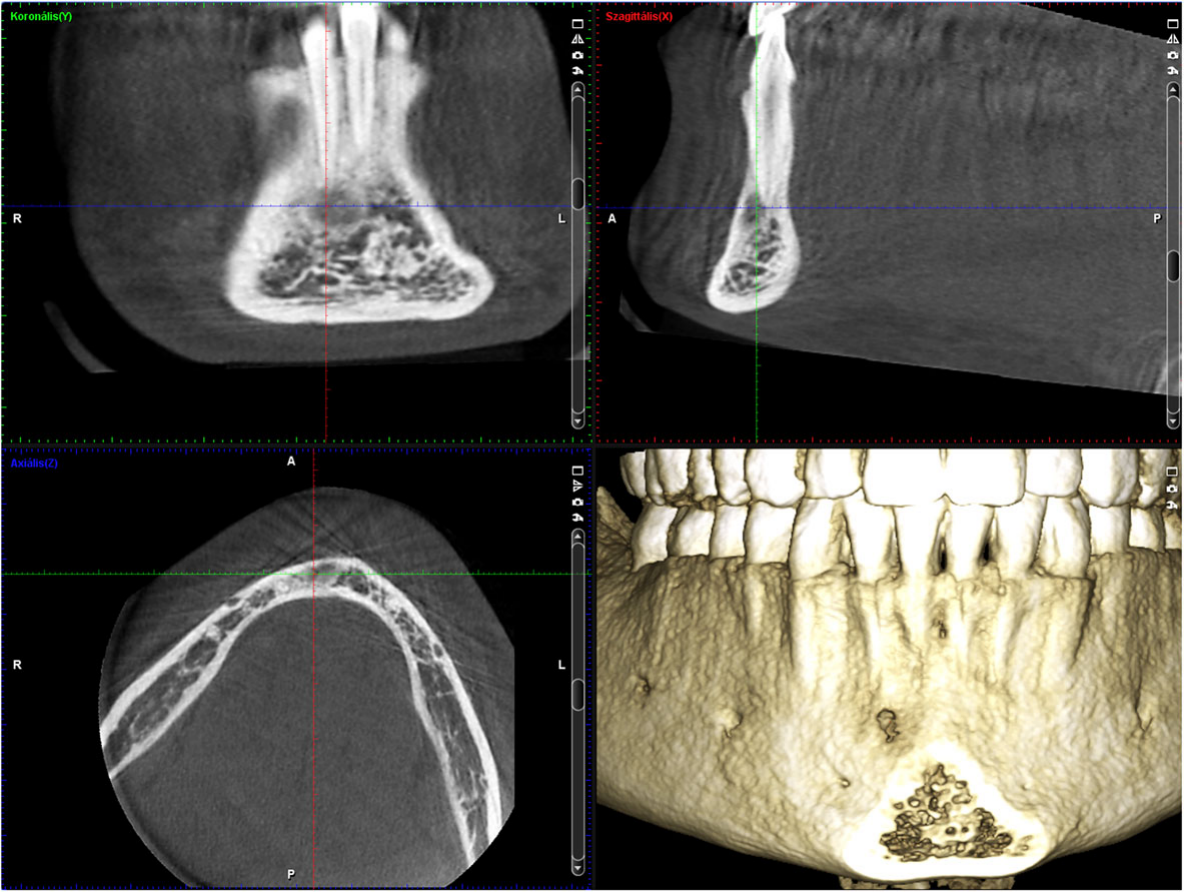
Early radiolucent lesion without radiopaque area were revealed around the root apex of 42 tooth on CBCT sections of patient 2. The inner surface of the buccal cortical begun to be eroded by the lesion. This is obvious when one compares this with the contralateral side

The 19-year-old Caucasian woman is the daughter of the first patient. CBCT showed a radiolucent lesion around the root of the lower right incisor (Fig. 3). She is presently asymptomatic after a one-year follow-up, but she occasionally felt tension and moderate pain in the right side of the mandibular region eradiating to her ear, approximately 2 years earlier. The symptoms had no dental background, and ceased gradually.

### Clinical examination

Pulp tests and periapical pathology diagnoses were made by authors on teeth 33,32,41,43 with the use of percussion, palpation and sensibility test.

### Radiological examination

Panoramic radiograph and CBCT scans of the first patient showed radiolucent lesions located in the periapical bone, specifically on lower incisors and canines – in the premandibular, and in the right molar region of the mandible with radiopaque parts showing the lesions inside (Figs. 1 and 2). The affected teeth were asymptomatic, CBCT proved the presence of apical pathology. The buccal cortical involvement was discovered with the aid of CBCT, which also affected the treatment plan because 2D imaging methods provide no information on bucco-lingual dimension. Prior to the surgical intervention the use of CBCT was motivated by the fact that only 3D imaging could ascertain the precise shape, location and involvement of surrounding anatomical landmarks of the lesion.

**Fig. 4:**
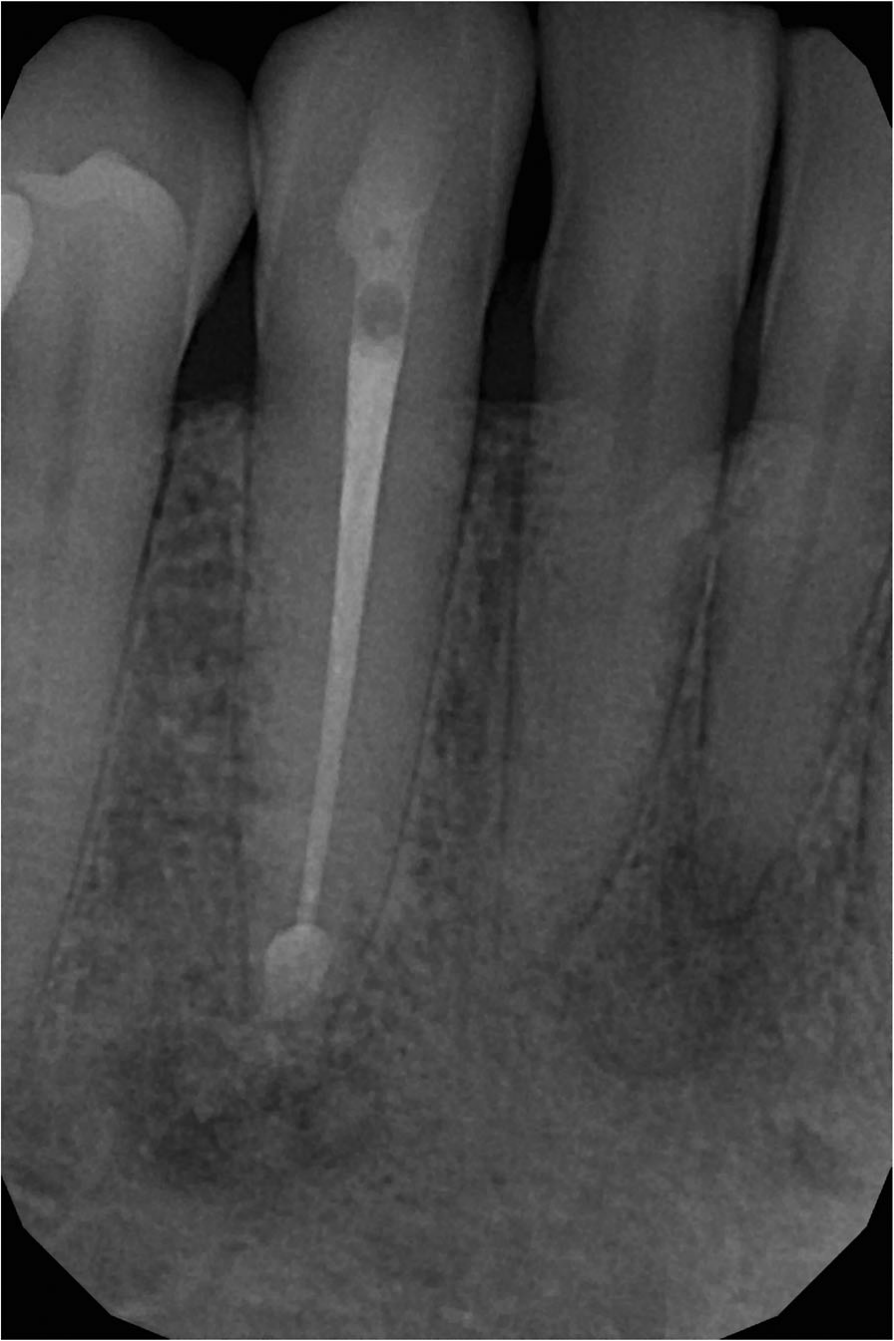
On the apex of the first patient’s lower canine a mixed (opaque and transparent) lesion is visible. Opaque focuses refer to remnants of the implanted bone graft material

**Fig. 5:**
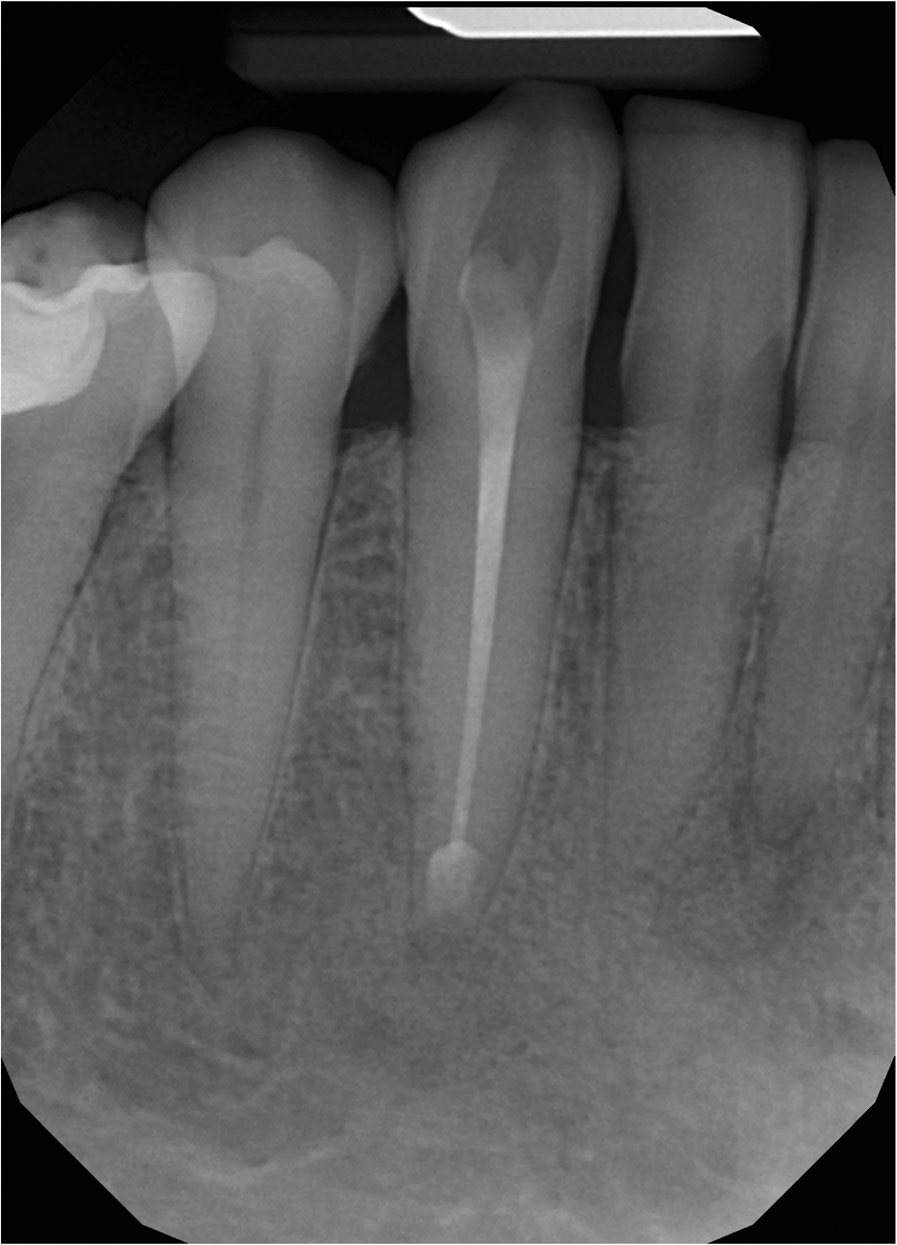
At the 6-month follow-up of the first patient, a homogenous area is visible inside the lesion with a thin radiolucent margin

**Fig. 6:**
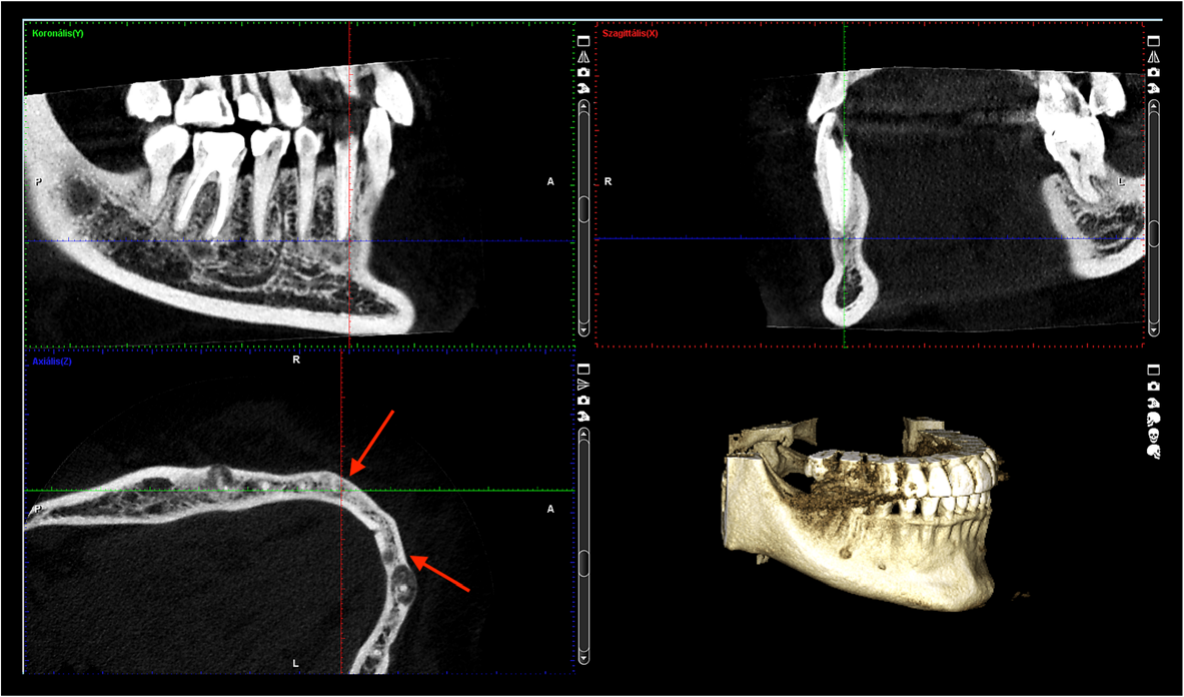
Eighteen months follow up CBCT image of patient 1. Buccal cortical of lower right canine has regenerated. Swelling of this area disappeared. Periapical new bone showing lacunas and wider trabeculas filling the previous volume of pathology

Figure 4 shows the postoperative condition (Fig. 4). At the 6-months periapical follow-up, the bone healing is being processed (Fig. 5). 18 months later it can be seen on the CT image that the buccal cortical bone surrounding the root of tooth 43 has regenerated (Fig. 6). In the case of the second patient, there were no notable changes in the radiographic status 1 year later, and she has no complaint at present.

### Histopathology

**Fig. 7:**
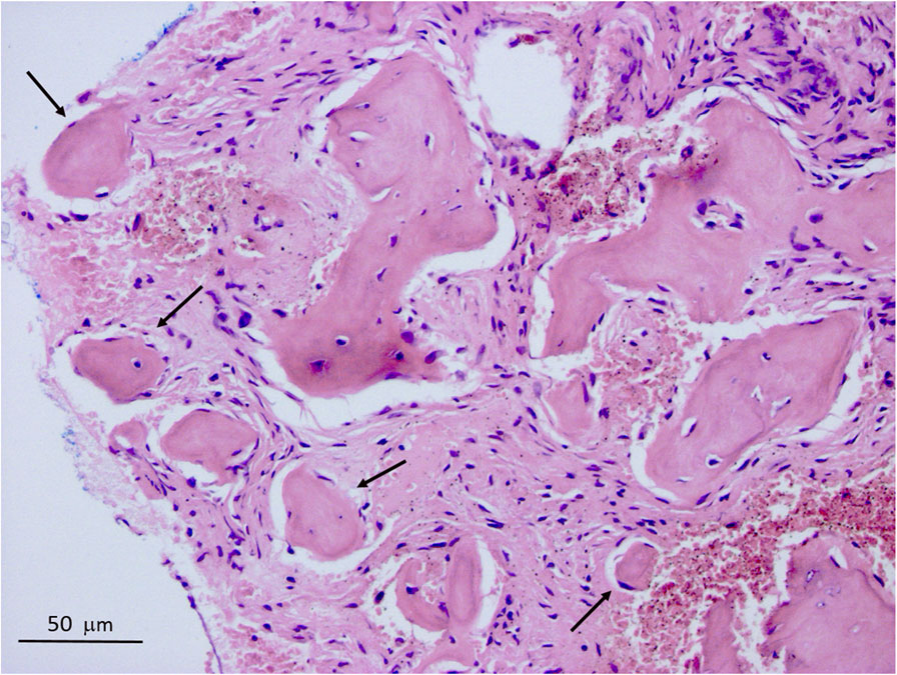
Histological image of the COD lesion of the first patient. Acellular fragments of cementum-like substances (arrows) in loose fibro-collagenous stroma. Unmarked lamellar bony formations show no osteoclastic activity. (HE, × 400)

The removed tissue pieces contained both connective tissue and calcified areas. In the calcified area, beside the irregular trabecular – lamellar bony formations, oval and globular cementum-like structures were present. In the non-calcified area connective tissue was found and connective tissue filled out the centre of another bony sample as well. No elements or remnants of a capsule were visible. The histological diagnosis was cemento-osseous dysplasia (Fig. 7).

## Discussion and conclusions

COD is a sporadically occurring benign lesion in the tooth-bearing areas characterized by fibrosus dysplasia [6] .Familial occurrence of the disease is very rare [13], its characteristics differ from non-hereditary cases by its occurrence also in younger ages [14], and is not connected to the female gender and black skin colour. All familial cases are grouped in the florid group, and shows autosomal familial inheritance [2, 15–17].

The condition is usually asymptomatic, discovered in routine panoramic radiographs, and, in most cases, no treatment is necessary. Apart from certain cases of the florid form, lesions have limited growth potential. The progress of the disease can be divided into three stages: osteolytic, mixed and matured osteogenic [4].

In radiologic imaging COD can easily be confused with inflammatory periapical processes. The typical appearance is a mineralized area visible within the radiolucent lesion. The lesion usually goes through a maturation process, which results in an increasing number of radiopaque areas, until finally a no longer growing radiopaque area with a lobular, ginger root-like appearance remains. In the mixed and densest stage, a radiolucent border separates it from the surrounding healthy bone [9, 18].

Histologically, all forms of COD have similar appearance; the lesion is not surrounded by a capsule, and, in the early, osteolytic stage it consists of fibrotic tissue rich in cells and vessels, from which cement-like deposits are absent. As the maturation progresses over time, cement-like formations and irregular trabeculae appear. In the final osteosclerotic stage, these structures connect to each other, and few cellular elements are visible [10, 19].

Differential diagnosis should be made between COD and the different forms of periodontitis in order to avoid unnecessary endodontic interventions [4, 20]. In the present case, the primary false diagnosis of the apical translucency around the lower right first molar resulted in an unnecessary root canal treatment, since the pulp was vital. Thereafter, the oral diagnostic staff was involved in defining the adequate diagnosis in order to prevent further unnecessary endodontic treatments of the anterior teeth. The present case raises the question of how many apical transparencies are misdiagnosed in general. Several cases were reported [21, 22] on inadequate treatment with COD, however, these studies do not provide numbers or proportions of misdiagnosis.

Distinguishing from Paget’s disease may also cause problems in the early stage. The microscopic image of Paget’s shows the characteristics of fibro-osseous dysplasia [23]. COD may also be confused with *fibrosus dysplasia* – the distinction may be based on the differences of the mineralized matrix, the significant face asymmetry and the developing malocclusion. COD has recently been differentiated from *cemento-ossifying fibroma*, where the histological image is similar, but the lesion is encapsulated and clearly separated from the surrounding healthy bone tissue. The option of *cementoblastoma* also occurs – it always connects to the root apex and is also a tumour [24]. In the case of *chronic sclerosing osteomyelitis*, differentiation may be made based on the lack of inflammatory signs [19, 25, 26].

Surgical treatment is limited for patients with severe clinical symptoms including pain and paraesthesia, those with expansive growth of the lesion, and candidates for dental implantation at the affected area [8, 19].

Terminology of COD has varied over the years. These changes depend on whether the focus is on the origin or the clinical presentation. In the latest WHO classification [10] three clinical forms of cemento-osseous dysplasia are distinguished:

### Periapical cemento-osseous dysplasia

It connects to the root apex region of the premandibular teeth, affects one or more teeth and is usually asymptomatic. It often leads to a false diagnosis, which may cause unnecessary intervention [4, 27].

### Focal cemento-osseous dysplasia

It is linked to one tooth, appears in the molar region in the tooth-bearing or edentulous area [6].

### Florid cemento-osseous dysplasia

The term was introduced by Melrose [9] who described the lesion in 1976. Authors agree that it is inherited as autosomal dominant trait [16]. It can occur in any quadrant of the maxilla and mandible as a multifocal multiquadrant lesion, it is often expansive and may rarely cause the deformation of the jaw bone. All types of COD but most often the florid form can cause changes in the blood supply of the bone: therefore, it increases the risk of infections (e.g. chronical osteomyelitis) in connection with surgical interventions – tooth removal in the affected area, implantation and biopsy – and may lead to the development of chronic osteomyelitis [28, 29]. Most often, the existence and expansion of this form cause pain. Paraesthesia has also been reported [2], which was the leading symptom of our first patient.

In the previous publication of the WHO [30] *familial gigantiform cementoma* is mentioned as the other expansive form of osseous dysplasia, which also shows autosomal dominant inheritance [17]. This type occurs in young people and causes significant swelling of the mandible [31]. In the 2017 WHO publication however, it is mentioned as an individual disease [10].

Just like some authors, such as Noffke places emphasis on the fact that the nomenclature should reflect the real nature of the lesion and recommends a different classification that takes both the clinical and biological characteristics into account. According to Noffke’s experience, the epicentre of all lesions was in the periapical region, but none of them was related to the surface of the tooth root: therefore, she suggests that the word “cementum” should be omitted. In her opinion, the term “periapical cemento-osseous dysplasia” is also unnecessary, as the other subgroups also originate from the periapical region. Based on the growth potential she has proposed the following classification: *expansive* and *non-expansive*, and within the non-expansive group a distinction between florid, focal and anterior mandibular form should be made. Finally, the expansive osseous dysplasia should be divided into familial and non-familial groups [32, 33].

The importance of our case is that it is a rare hereditary form of COD. Two regions were affected in the mother, the anterior and the right posterior regions. The diagnosis of COD was confirmed by histology. Based on the clinical characteristics, the case was classified as florid COD. However, if we examine only the daughter, then the diagnosis would be periapical COD, because the pathology developed in the anterior region only. As familial inheritance was described associated only with the florid form, we must classify the second patient as belonging to the florid group according to the present classification. This inconsistency raised the possibility that the periapical and focal form could in fact be the initial or unifocal manifestation of the florid COD [26, 34, 35]. Neither the periapical nor the focal version turns necessarily in to the florid, multiplex disease: moreover, these can recover at any stage. To date no histological differences have been reported distinguishing these forms, and all the COD variants have the same microscopic features [10, 21]. In the case of the daughter, a long-term follow-up will show whether further lesions will develop in other regions or not, which would mean that one COD form could turn into another. We agree with Summerlin who pointed out the possibility in his study that the single or initial condition may develop into the florid form [26]. For this reason, we think that the new classification proposed by Noffke is well established and more helpful for build-up treatment protocols: *Raubenheimer, Noffke: “Dividing the OD’s in non-expansive- (conventional, with no- or minimal expansion and generally occurring in patients above the 3th decade of life) and expansive subtypes. This division provides clear guidelines on management, as the approach to the treatment of the expansive group is surgery and the non-expansive types require no treatment unless infected”* [34]. We might consider using only the focal term instead of the focal and periapical because “both are the same entity with different locations” [35, 36]. It is based on the fact that the origin of all subtypes of COD is the same - the cells of periodontal ligaments [1–3], so we have to divide the different forms not on the basis of the anatomical location but the clinical and genetic features. The early stage and familial appearance of COD cases presented in this study serve as an example that the different forms of the recent classification cannot be used strictly.

Cemento-osseous dysplasia is a rare benign fibro-osseous disease of the jaws associated with vital teeth and generally no need for intervention, but follow-up is recommended. The differential diagnosis between the early stage of this fibro-osseous lesion and periapical inflammations and cysts is difficult to make but it is very important in order to avoid unnecessary endodontic treatments. On the other hand, we are faced with the problem that the current categories do not cover the early stage of a hereditary, possible florid form of cemento-osseous dysplasia. Familial occurrence of COD is very rare, and the previous reports found exclusively the florid subtype in all family members. In contrast, in the present report cemento-osseous dysplasia diagnosed in the mother and her daughter were of different subtypes. Our cases attract attention to the fact that the age range given in the literature is a mean calculated based on the time of recognition of the disease, and thus does not always follow the dinamics of the pathogenesis. Due to the fact that in the preliminary stages COD resembles periapical inflammation, therefore very few people diagnose it as COD. It is pertinent to control family members as well, and if a lesion is present make them aware of the implications.

## Data Availability

All data generated or analysed during this study are included in this published article [and its supplementary information files].

## Abbreviations

CBCT: Cone Beam Computed Tomography
COD: Cemento-osseous dysplasia
WHO: World Health Organization
